# Commentary: Dopamine-Dependent Early Synaptic and Motor Dysfunctions Induced by α-Synuclein in the Nigrostriatal Circuit

**DOI:** 10.3389/fnagi.2021.790224

**Published:** 2021-11-29

**Authors:** Jiahui Zhang, Heng Liu, Hong Jiang

**Affiliations:** Department of Physiology, Shandong Provincial Key Laboratory of Pathogenesis and Prevention of Neurological Disorders and State Key Disciplines: Physiology, School of Basic Medicine, Medical College, Qingdao University, Qingdao, China

**Keywords:** Parkinson’s disease, substantia nigra, motor function, α-synuclein, nigrostriatal circuit

## Introduction

Parkinson’s disease (PD) is a neurodegenerative illness that causes severe motor dysfunction in individuals. Symptoms of PD include motor retardation, uncontrollable tremors at rest, postural problem, and stiffness (Surmeier, 2018; Ganguly et al., 2021), as well as a variety of non-motor symptoms (Holanda et al., 2021). The predominant neuropathologic hallmarks are synaptic damage and loss of dopamine (DA) neurons in the substantia nigra pars compacta (SNpc) (Trudler et al., 2021). Although PD is currently incurable, medication can help to improve symptoms and maintain the quality of life (Prasad and Hung, 2021). The buildup of α-synuclein (α-syn) is a known etiology of PD pathogenesis (Zhang et al., 2018). In the early stages of PD, α-syn accumulation impairs synaptic vesicle fusion and aggregation (Longhena et al., 2017), affecting neurotransmitter release and ultimately leading to nigrostriatal neuronal death (Imbriani et al., 2018). It has been demonstrated that α-syn preformed fibers (α-syn-PFF) directly injected in cell culture or rodent brain can bind to endogenous α-syn (Volpicelli-Daley et al., 2011), producing pathological inclusions and/or reproducing PD features. Although the early diagnosis of PD is essential in the treatment of patients, the mechanism of early pathogenesis still needs to be further explored.

## Substantia Nigra Pars Compacta Is Retrogradely Affected by Affecting Its Dopaminergic Neurons and Synaptic Plasticity

Previously, Hijaz et al. used animal models to study the key role of α-syn in the pathology of PD, suggesting that α-syn can be transmitted between neurons (Luk et al., 2009; Hijaz and Volpicelli-Daley, 2020). In this study (Tozzi et al., 2021), how α-syn induces changes in synaptic properties and motor dysfunction in a time-dependent manner in the nigrostriatal circuit was further explored. Stoyka et al. have reported that intrastriatal injections of α-syn-PFF can mimic various features of rodent PD pathology, such as reduced DA in the SNpc, α-syn aggregation, and diffusion between brain regions directly associated with the injection site (Luk et al., 2012; Koprich et al., 2017; Patterson et al., 2019; Stoyka et al., 2020), and the appearance of Lewy bodies, resulting in neuronal reduction and death (Gallegos et al., 2015; Wong and Krainc, 2017). Because of the predominance of α-syn lesions at the synaptic terminus, early synaptic damage precedes axonal degeneration, which is followed by retrograde progression (Chandra et al., 2005; Calo et al., 2016; Wu et al., 2019).

Based on the above research, this study (Tozzi et al., 2021) identifies two early time points, 6 and 12 weeks after α-syn-PFF injection, to further clarify how intrastriatal injection of α-syn-PFF in rodents induces time-dependent motor behavior and different forms of alterations in cortical striatal plasticity, such as long-term potentiation (LTP) and long-term depression (LTD), by affecting the firing frequency of DA neurons in the SNpc region. In line with previous studies (Luk et al., 2012), a significant decrease in striatal DA release and mild motor behavioral changes were already present at 12 weeks after α-syn-PFF injection. In addition, the model suggested by Tozzi et al. (2021) embodies more novel features in some aspects, such as a reduced striatal TH immunoreactivity at 6 weeks after α-syn-PFF injection, whereas Luk et al. only reported similar changes after 24 weeks. The results of this study found that the retrograde translocation of labeled phosphorylated-alpha-synuclein (p-α-syn) occurred in the SNpc region at both 6 and 12 weeks after striatal injection and that DA neuron activity was altered in a time-dependent manner. The number of DA neurons decreased after 6 weeks of injection and the frequency of spontaneous firing was reduced, whereas an increase in firing frequency was found after 12 weeks of injection and this alteration was specific. This strange phenomenon is thought to be a form of “stressful pacemaking” before the massive death of DA neurons. Synaptic plasticity was also altered following striatal injection. For example, impairment of LTP was found at both 6 and 12 weeks of injection, but LTD was not impaired until 12 weeks postinjection. For this result, Tozzi et al. concluded that the synaptic damage was the result of a decrease in DA release leading to a decline in endogenous cannabinoid release, which acted in a retrograde manner on glutamatergic terminals, leading to an excessive enhancement of glutamate activity and mediating excitatory neurotoxicity. In assessing changes in motor function, it was found that in the grid-walking task, a prolongation of latency and resting period of climbing in rats occurred at 12 weeks after striatal injection but not at the 6-week mark. However, in the open field apparatus, a reduction in time in the open field center was seen at both 6 and 12 weeks after striatal injection compared with controls, possibly due to the different sensitivity of these two methods of detecting motor dysfunction. Interestingly, not all substantia nigra areas were subjected to retrograde damage by striatal injection, as the spontaneous firing frequency of neurons in the substantia nigra pars reticulata (SNpr) area was not affected. The findings obtained from the analysis of the early stages of the disease can improve our understanding of the different susceptibilities to disease in different ganglion regions.

Furthermore, it is extremely intriguing that this study explores, for the first time, the role of impairment of the α-syn-dependent dopaminergic nigrostriatal system in the onset and development of deficits in striatal synaptic properties and plasticity, which are associated with autonomic motor dysfunction. This study contributes to further understanding of the mechanisms of α-syn-PFF synaptic and basal ganglia plasticity in PD. Also, it was found that the common drug L-Dopa treats α-syn-PFF-induced motor dysfunction, striatal synaptic plasticity impairment, and reduced spontaneous excitatory synaptic current. It suggests that α-syn-PFF-induced dysfunction of the DA system plays a key role in the early stages of PD. This research may also be transformed into clinical trials to provide a basis for early conditioning therapies for PD (Luk et al., 2012; Dawson and Dawson, 2019).

There is growing evidence that α-syn-induced synaptic dysfunction plays an important role in the occurrence and development of PD. Previously, the team of Trudler, D., demonstrated that α-syn can lead to synapse loss (Trudler et al., 2021). They found that oligomeric α-syn can lead to the release of glutamate from astrocytes, which over activates NMDA receptors, resulting in synaptic damage by patch clamp. Therefore, investigating the mechanisms by which α-syn causes early synaptic damage can help us to better understand and develop new therapeutic targets and diagnostic markers for PD. In this study (Tozzi et al., 2021), the use of L-Dopa to treat synaptic dysfunction caused by a striatal injection of α-syn-PFF provides a potential target for early-stage PD treatment.

## Conclusion

Taken together, this is the first report explaining how α-syn-PFF affects DA neuronal function in the substantia nigra by retrogradation in the striatum, with important implications for the early detection and treatment of PD. The differences that emerge in motor function testing also suggest that motor dysfunction may be difficult to identify and assess in the early clinical stages of PD due to powerful compensatory mechanisms. Although it has been demonstrated experimentally that α-syn-PFF can further induce impairment in synaptic LTP and LTD by affecting the firing of DA neurons in the early stages of PD, there is still a long way to go to apply the detection of such early pathological changes to the clinic so that PD becomes capable of early detection, early intervention.

## Author Contributions

JZ and HL conceived the article and wrote the first draft. JZ, HL, and HJ reviewed and revised the manuscript. All authors contributed to the article and approved the submitted version.

## Conflict of Interest

The authors declare that the research was conducted in the absence of any commercial or financial relationships that could be construed as a potential conflict of interest.

## Publisher’s Note

All claims expressed in this article are solely those of the authors and do not necessarily represent those of their affiliated organizations, or those of the publisher, the editors and the reviewers. Any product that may be evaluated in this article, or claim that may be made by its manufacturer, is not guaranteed or endorsed by the publisher.
